# Fístula Coronário-Cavitária, uma Causa Incomum de Insuficiência Cardíaca

**DOI:** 10.36660/abc.20240809

**Published:** 2025-03-27

**Authors:** Hernán Patricio García Mejía, Matheus Carvalho Alves Nogueira, Bruno Mahler Mioto, Nilson Tavares Poppi, Luiz Antonio Machado Cesar, Luhanda Leonora Cardoso Monti Sousa, Luís Roberto Palma Dallan

**Affiliations:** 1 Hospital das Clínicas Faculdade de Medicina Universidade de São Paulo São Paulo SP Brasil Instituto do Coração do Hospital das Clínicas da Faculdade de Medicina da Universidade de São Paulo, São Paulo, SP – Brasil

**Keywords:** Isquemia Miocárdica, Angiografia Coronária, Fístula, Insuficiência Cardíaca

## Introdução

As fístulas coronárias (FCs), são definidas como uma conexão anômala direta entre uma ou mais artérias coronárias com um grande vaso ou câmara cardíaca. A maior parte das FCs drenam em um sistema de menor pressão, como ventrículo direito (VD) 40%, átrio direito (AD) 26%, artéria pulmonar (AP) 17%, seio coronário 7% e vena cava 1%. A apresentação clínica dependerá da localização e volume do *shunt* gerado pela fístula. Aproximadamente 50% dos casos são assintomáticos e, quando presentes, podem incluir dispneia, palpitações, dor torácica ou, em quadros mais graves, insuficiência cardíaca (IC), síndromes coronarianas agudas e arritmias.^1,2^

### Relato de Caso

**Figura 1:**
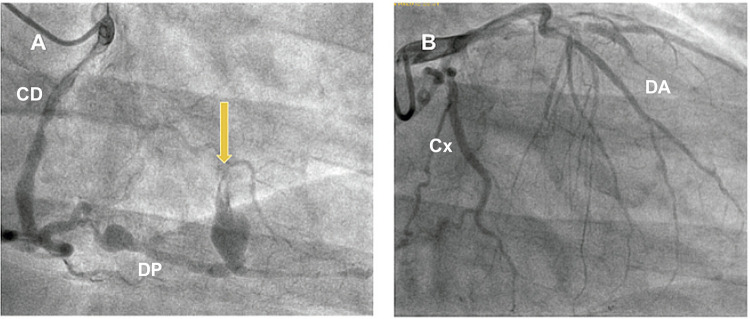
– A) Artéria coronária direita (CD), evidência de lesão grave da artéria descendente posterior (DP) recebendo ramo colateral da artéria circunflexa (Cx) e presença de fístula coronária-cavitária (seta amarela) para o ventrículo direito. B) Artéria coronária esquerda. DA: artéria descendente anterior.

Homem de 63 anos, com antecedente de hipertensão arterial sistêmica (HAS), diabetes mellitus (DM) não insulino-requerente, doença renal crônica (DRC) não-dialítica, dispneia aos moderados esforços, angina CCS 2 com diagnóstico de doença arterial coronariana (DAC) e achado incidental de fístula coronário-cavitária de DP para VD em 2012 na coronariografia (Figura 1). Cirurgia de revascularização do miocárdio (CRVM) no mesmo ano, com artéria torácica interna esquerda (ATIe) *in situ* para artéria descendente anterior (DA); veia safena (Sf) para artéria descendente posterior (DP), Sf para primeiro ramo marginal esquerdo (MgE1). Foi realizado ECOTT na ocasião com evidência de fração de ejeção do ventrículo esquerdo (FEVE) de 43%.

Após sete anos, evoluiu com angina CCS 3, dispneia aos pequenos esforços, ortopneia, dispneia paroxística noturna e edema de membros inferiores, a despeito de terapia medicamentosa otimizada para IC.

Ecocardiograma em 29/03/2022 mostrava um átrio esquerdo (AE) aumentado (60 mm), FEVE de 30%, acinesia de septo e hipocinesia dos demais segmentos miocárdicos, disfunção de VD e disfunção diastólica grau 4. Deu entrada no Pronto Socorro do Instituto do Coração (InCor) do Hospital das Clínicas da Universidade de São Paulo (HCFMUSP) em 06/05/2022, com quadro compatível de insuficiência cardíaca descompensada perfil B. Realizada compensação clínica e encaminhado para acompanhamento ambulatorial.

Primeira consulta ambulatorial em 03/06/2022, com manutenção dos sintomas e sinais de congestão pulmonar e sistêmica ao exame físico. Realizada otimização da terapia medicamentosa, orientado restrição hídrica e solicitados exames complementares.

No ECOTT realizado em 25/07/2022, mantendo as alterações prévias e uma FEVE 36%, insuficiência mitral moderada com *tethering* de cúspide anterior, insuficiência tricúspide moderada e valva pulmonar com sinais indiretos de hipertensão pulmonar (PSAP 53 mmHg), não sendo descrita a presença de fluxos na parede livre do VD.

Realizada primeira reunião de *Heart Team* no dia 09/09/2022. Por se tratar de um paciente com CRVM prévia, DRC, piora clínica e da função ventricular, cuja causa poderia estar atribuída à evolução da doença coronária, à patência dos enxertos, bem como ao aumento do débito da fístula, e devido à inexistência de estudo anatômico nos últimos 10 anos, decidiu-se por realizar novo cateterismo cardíaco.

**Figura 2:**
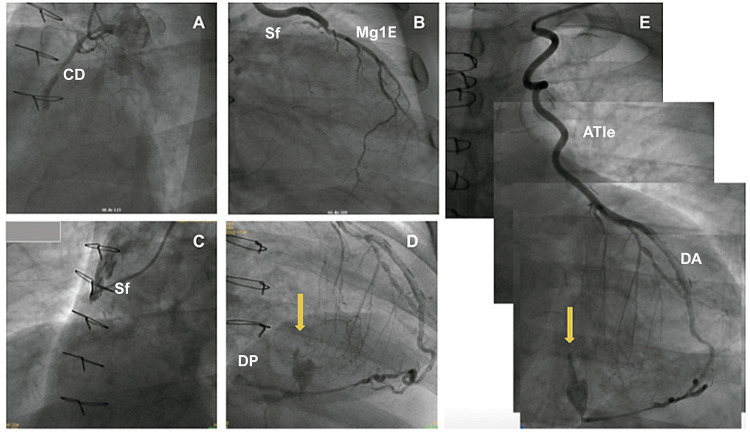
– A) Artéria coronária direita (CD): Lesão ocluída 100% no terço médio; B) Ponte de safena para primeiro ramo marginal esquerdo (MgE1) sem lesões obstrutivas; C) Ponte de Safena para artéria CD com lesão de 100% no óstio; D) Fluxo proveniente da DP; E) Fístula (seta amarela), dependente de fluxo da ATIe-DA. Enxerto de artéria torácica interna esquerda (ATIe) para artéria descendente anterior (DA) sem lesões obstrutivas.

Cateterismo realizado em 20/10/2022, com evidência de, DA ocluída no terço proximal; Artéria circunflexa com lesão segmentar de 60% no terço médio; MgE1 com lesão focal de 70% no terço proximal, e competição de fluxo a partir do seu terço médio; CD com oclusão de 100% no terço médio; ponte de veia safena para CD com lesão de 100% no óstio; enxerto de ATIe para DA e ponte de veia safena para MgE1 sem lesões obstrutivas. (Figura 2).

Realizada segunda reunião de Heart Team em 20/12/2022 e, após avaliação do cateterismo, optado inicialmente por fechamento percutâneo da fístula. Porém, devido ao alto risco de fechamento da anastomose da ATIe com DA durante o procedimento, o mesmo não foi efetuado. Rediscutido em terceira reunião em 24/02/2023, sendo optado por tratamento cirúrgico de fechamento da fístula com ecocardiograma transesofágico (ECOTE) para guiar procedimento.

**Figura 3:**
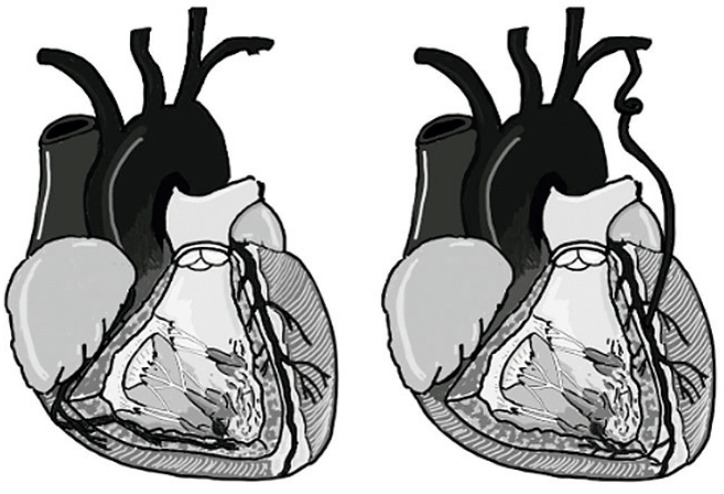
– Desenho esquemático da origem do fluxo da fístula antes e depois da CRVM para o VD. CRVM: cirurgia de revascularização miocárdica; VD: ventrículo direito.

Foi realizado procedimento cirúrgico no dia 16/03/2023. O ECOTE pré-circulação extracorpórea (CEC) observou fluxo diastólico de baixa velocidade na parede livre do VD, sugestivo de fístula coronário-cavitária com múltiplas origens em região sub valvar tricúspide, no terço médio e em região apical. Durante a cirurgia foram identificadas quatro pontes: ATIe-DA pérvia, Saf-MgE1 pérvia, Saf-DP fechada, Saf-Diagonalis fechada. Realizada atriotomia direita e exploração do VD através da valva tricúspide com presença de fluxo arterial para o VD, com origem provável da ATIe (a aorta ascendente estava clampeada). Identificada DA na região apical e os ramos da coronária direita, realizada rafia dos ramos da CD desde a porção distal até o começo da ponte de safena para VD (incluindo a ponte fechada na rafia). Reavaliação do VD, com redução do fluxo, mas sem cessamento total. Realizada ligadura da DA após a curva na região apical, porém ainda assim mantendo um pequeno fluxo para o VD, tendo que ser retirados os pontos na DA e optado por encerrar o procedimento pela redução do fluxo. Novo ECOTE mostrou persistência de pequenos fluxos sugestivos de fístulas coronário-cavitárias (Figura 3).

No pós-operatório, o paciente evoluiu com necessidade de hemodiálise. Nos dias seguintes, apresentou melhora progressiva dos sintomas anginosos e de IC, associado a maior tolerância aos exercícios e à fisioterapia.

Ecocardiograma realizado no dia 14/11/2023 com redução das dimensões do AE, e do VE, hipertrofia concêntrica de grau moderado, FEVE 42%, às custas de acinesia dos segmentos médio e basal da parede ínfero-septal e acinesia da parede inferior. O VD apresentava hipocinesia discreta, insuficiência mitral discreta e insuficiência tricúspide moderada e diminuição da hipertensão pulmonar (PSAP 39 mmHg).

Em nova consulta de controle após 18 meses da internação (10/2024), paciente em CF I, sem intercorrências no período, mantendo diálise trissemanal e sem sinais congestivos ao exame físico e melhora da qualidade de vida.

## Discussão

As FCs, consideradas anomalias raras, afetam aproximadamente 0,1% a 0,2% da população,^3^ com uma incidência exata ainda desconhecida. A maioria das FCs podem se manter assintomáticas ou fechar espontaneamente ao longo da vida, existindo uma alta taxa de casos não diagnosticados. As principais causas são congênitas, traumáticas e adquiridas devido ao advento de técnicas cirúrgicas, endovasculares e implante de dispositivos. As FCs podem-se classificar em pequenas, intermediárias e grandes se o diâmetro da fístula for <1, ≥1 - 2, ou >2 vezes o diâmetro maior do vaso coronário que não alimenta a fístula respectivamente.

O diagnóstico das FCs a maioria das vezes se dá como um achado incidental após a realização de um exame de imagem, como neste caso. Atualmente a angiografia tem se tornado o padrão-ouro no diagnóstico das FCs apesar das suas limitações como a sobreposição de imagens. Existem outros métodos como o ecocardiograma e angiotomografia que auxiliam no pré, pós-procedimento e programação da abordagem.^4-6^

A repercussão clínica das FCs depende: 1) da sua localização - geralmente, fístulas proximais tendem a ter um fluxo maior que as fístulas distais; 2) do calibre da fístula – calibres maiores podem gerar fenômenos de roubo coronário; 3) do local de drenagem - usualmente, as fístulas desembocam em lugares de menor pressão, com *shunt* e sobrecarga de volume da câmara ou grande vaso receptor,^7^ tornando-se em indicações da necessidade de abordagem. A atualização de 2018 da diretriz do *American College of Cardiology/American Heart Association*, enfatiza a importância da avaliação em *Heart Team* sobre o viabilidade e escolha da abordagem em centros com experiência em técnicas de fechamento, tanto cirúrgicas como percutâneas.^8^

Fatores anatômicos como a localização (proximal ou distal) influenciam a escolha do método mais adequado de abordagem das FCs. Fístulas do leito coronariano distal, como no caso do nosso paciente, apresentam maior risco de complicações no tratamento endovascular além da presença de uma área maior em risco, sendo recomendada uma abordagem cirúrgica.^6^

Neste contexto particular, um paciente previamente diagnosticado com DAC, apresentando alterações na motilidade segmentar e a descoberta incidental de uma fístula coronário-cavitária durante a coronariografia, presumivelmente de etiologia congênita e cujo fluxo principal era derivado da coronária direita, manifestou sintomas evidentes e progressivos de IC, possivelmente decorrentes da progressão ou coexistência das doenças.

O caso apresentado mostra-se desafiador sob alguns aspectos: primeiramente, quanto ao diagnóstico da etiologia da IC pela difícil caracterização nos métodos de imagem não invasivos habituais como o ECOTT. Apesar de o ECOTE possuir maior sensibilidade para a detecção de fluxos, local de entrada e terminação do *shunt*, o tamanho e características anatômicas da fístula, neste caso, tiveram que ser estabelecidos por meio da angiografia, preferida neste caso visto as comorbidades do paciente e por ser considerada o padrão-ouro; quanto ao tratamento, devido ao alto risco de complicações durante os procedimentos, localização da fístula, miocárdio em risco, ausência de evidências robustas no tratamento percutâneo em pacientes com revascularização prévia foi optado por uma abordagem cirúrgica; neste caso, após a realização do procedimento cirúrgico, o paciente evoluiu de maneira favorável ao longo dos meses posteriores, com remodelamento das câmaras esquerdas, diminuição da PSAP de 53 para 39 mmHg e melhora clínica, atualmente em classe funcional I. Embora as fístulas coronarianas sejam uma etiologia pouco frequente, muitas vezes desconhecida de IC, a correção, mesmo em ocasiões tardias, como no caso em questão, pode levar a uma melhora do prognóstico e qualidade de vida dos pacientes.

## Conclusões

As FCs continuam sendo uma entidade de difícil diagnóstico devido à sua baixa incidência, grande parte das vezes se mantendo clinicamente assintomáticas até a vida adulta e como visto neste relato, de natureza dinâmica, quando sintomáticas, podem levar a estados de sobrecarga hemodinâmica que, concomitantemente com outras patologias, dificultam o manejo desses pacientes.^9-11^

A escassez de dados na literatura a respeito do tema dificulta não só o diagnóstico das FCs como, acima de tudo, seu tratamento, especialmente com relação a quando e como tratar. Essas questões, provavelmente, dependem de inúmeros fatores, e devem ser avaliadas caso a caso. Não é possível afirmar, por exemplo, com base nos dados que temos disponíveis, se a abordagem cirúrgica da fístula deveria ter sido feita no momento da CRVM em 2012, ou se foi correto manter conduta expectante àquela época. Apesar disso, na ausência de um grupo controle, a avaliação prospectiva do paciente deixa clara a melhora apresentada pelo paciente após o procedimento realizado em 2023, em termos clínicos e de imagem, pelo menos no curto tempo de seguimento avaliado.
