# Supplementary material for: Fístula Coronário-Cavitária, uma Causa Incomum de Insuficiência Cardíaca
Source: Arq Bras Cardiol. 2025 Mar 27;122(3):e20240809. [Article in Portuguese] doi: 10.36660/abc.20240809 (PMC12058156; doi:10.36660/abc.20240809)
Supplement: Translation

# Translation: Coronary-Cavitary Fistula, an Unusual Cause of Heart Failure

**DOI:** 10.36660/abc.20240809i

**Authors:** Hernán Patricio García Mejía, Matheus Carvalho Alves Nogueira, Bruno Mahler Mioto, Nilson Tavares Poppi, Luiz Antonio Machado Cesar, Luhanda Leonora Cardoso Monti Sousa, Luís Roberto Palma Dallan

**Affiliations:** 1 Brazil Instituto do Coração do Hospital das Clínicas da Faculdade de Medicina da Universidade de São Paulo, São Paulo, SP – Brazil

**Keywords:** Myocardial Ischemia, Coronary Angiography, Fistula, Heart Failure

## Introduction

Coronary fistulas (CFs) are defined as a direct anomalous connection between one or more coronary arteries with a large vessel or cardiac chamber. Most CFs drain into a lower pressure system, such as the right ventricle (RV) 40%, right atrium (RA) 26%, pulmonary artery (PA) 17%, coronary sinus 7%, and vena cava 1%. The clinical presentation will depend on the location and volume of the shunt generated by the fistula. Approximately 50% of cases are asymptomatic and, when present, may include dyspnea, palpitations, chest pain, or, in more severe cases, heart failure (HF), acute coronary syndromes, and arrhythmias.^1,2^

## Case Report

A 63-year-old man with a history of systemic arterial hypertension (SAH), non-insulin-requiring diabetes mellitus (DM), non-dialysis chronic kidney disease (CKD), dyspnea on moderate exertion, CCS 2 angina with a diagnosis of coronary artery disease (CAD) and an incidental finding of coronary-cavitary fistula from PD to RV in 2012 on coronary angiography (Figure 1). Coronary artery bypass grafting (CABG) in the same year, with the left internal thoracic artery (LITA) *in situ* to the anterior descending artery (AD); saphenous vein (Sf) to the posterior descending artery (PD), Sf to the first left marginal branch (MgE1). TTE was performed at the time with evidence of a left ventricular ejection fraction (LVEF) of 43%.

**Figure 1:** – A) Right coronary artery (RCA), evidence of severe injury to the posterior descending artery (PD) receiving a collateral branch from the circumflex artery (Cx) and presence of coronary-cavitary fistula (yellow arrow) to the right ventricle. B) Left coronary artery. AD: anterior descending artery.

After seven years, he developed CCS 3 angina, dyspnea on mild exertion, orthopnea, paroxysmal nocturnal dyspnea, and lower limb edema despite optimized drug therapy for HF.

An echocardiogram on 03/29/2022 showed an enlarged left atrium (LA) (60 mm), LVEF of 30%, septal akinesia and hypokinesia of the other myocardial segments, RV dysfunction, and grade 4 diastolic dysfunction. He was admitted to the Emergency Room of the Instituto do Coração (InCor) of the Hospital das Clínicas of the University of São Paulo (HCFMUSP) on 05/06/2022, with a picture compatible with decompensated heart failure profile B. Clinical compensation was performed and he was referred for outpatient follow-up.

First outpatient consultation on 06/03/2022, with maintenance of symptoms and signs of pulmonary and systemic congestion on physical examination. Optimization of drug therapy was performed, fluid restriction was advised, and additional tests were requested.

In the TTE performed on 07/25/2022, maintaining the previous alterations and an LVEF of 36%, moderate mitral regurgitation with anterior cusp tethering, moderate tricuspid regurgitation, and pulmonary valve with indirect signs of pulmonary hypertension (PAP 53 mmHg), with no description of the presence of flows in the free wall of the RV.

The first Heart Team meeting was held on 09/09/2022. As this was a patient with previous CABG, CKD, clinical worsening, and ventricular function, the cause of which could be attributed to the progression of coronary disease, graft patency, as well as increased fistula output, and due to the lack of anatomical study in the last 10 years, it was decided to perform a new cardiac catheterization.

Catheterization performed on 10/20/2022, with evidence of occluded LAD in the proximal third; Circumflex artery with 60% segmental lesion in the middle third; MgE1 with 70% focal lesion in the proximal third, and flow competition from its middle third; RC with 100% occlusion in the middle third; saphenous vein bridge to RC with 100% lesion in the ostium; LITA graft to LAD and saphenous vein bridge to MgE1 without obstructive lesions. (Figure 2).

**Figure 2:** – A) Right coronary artery (RCA): 100% occluded lesion in the middle third; B) Saphenous graft to the first left marginal branch (MgE1) without obstructive lesions; C) Saphenous graft to the RCA artery with 100% lesion in the ostium; D) Flow from the PD; E) Fistula (yellow arrow), dependent on flow from the LITA-AD. Left internal thoracic artery (LITA) graft to the anterior descending artery (AD) without obstructive lesions.

A second Heart Team meeting was held on 12/20/2022, and after evaluating the catheterization, it was initially decided to perform percutaneous closure of the fistula. However, due to the high risk of closing the LITA anastomosis with AD during the procedure, the procedure was not performed. It was discussed again at a third meeting on 02/24/2023, and surgical treatment to close the fistula with transesophageal echocardiography (TEE) to guide the procedure was chosen.

A surgical procedure was performed on 03/16/2023. Pre-extracorporeal circulation (ECC) TTE observed low-speed diastolic flow in the RV free wall, suggestive of coronary-cavitary fistula with multiple origins in the tricuspid subvalvular region, in the middle third and the apical region. During surgery, four bridges were identified: patent LITA-AD, patent Sf-MgE1, closed Sf-PD, and closed Sf-Diagonalis. Right atriotomy and exploration of the RV through the tricuspid valve were performed with the presence of arterial flow to the RV, with probable origin from the LITA (the ascending aorta was clamped). AD was identified in the apical region, and the branches of the right coronary artery and suture of the branches of the RC was performed from the distal portion to the beginning of the saphenous graft to the RV (including the bridge closed at the suture). Reassessment of the RV showed reduced flow but without total cessation. Ligation of the LAD was performed after the curve in the apical region, but still maintaining a small flow to the RV, and the stitches in the LAD had to be removed and it was decided to end the procedure by reducing the flow. A new TTE showed the persistence of small flows suggestive of coronary-cavitary fistulas (Figure 3).

**Figure 3:** – Schematic drawing of the origin of the fistula flow before and after CABG to the RV. CABG: coronary artery bypass grafting; RV: right ventricle.

In the postoperative period, the patient required hemodialysis. In the following days, he showed progressive improvement in angina and HF symptoms, associated with greater tolerance to exercise and physiotherapy.

An echocardiogram performed on 11/14/2023 showed reduced LA and LV dimensions, moderate concentric hypertrophy, and LVEF 42% due to akinesia of the middle and basal segments of the inferoseptal wall and akinesia of the inferior wall. The RV showed mild hypokinesia, mild mitral regurgitation, moderate tricuspid regurgitation, and decreased pulmonary hypertension (PAP 39 mmHg).

In a new control consultation after 18 months of hospitalization (10/2024), the patient was in CF I, without complications during the period, maintaining triweekly dialysis and without signs of congestive disease on physical examination and improved quality of life.

## Discussion

CFs, considered rare anomalies, affect approximately 0.1% to 0.2% of the population,^3^ with an exact incidence still unknown. Most CFs can remain asymptomatic or close spontaneously throughout life, with a high rate of undiagnosed cases. The main causes are congenital, traumatic, and acquired due to the advent of surgical, endovascular, and device implantation techniques. CFs can be classified as small, intermediate, and large if the diameter of the fistula is <1, ≥1 - 2, or >2 times the largest diameter of the coronary vessel that does not feed the fistula respectively.

The diagnosis of CF is most often made as an incidental finding after an imaging exam, as in this case. Currently, angiography has become the gold standard in the diagnosis of CF despite its limitations, such as image overlap. There are other methods, such as echocardiography and angiotomography, that assist in pre- and post-procedure assessment and planning of the approach.^4-6^

The clinical repercussions of coronary fistulas depend on 1) their location - generally, proximal fistulas tend to have a greater flow than distal fistulas; 2) the caliber of the fistula - larger calibers can generate coronary steal phenomena; 3) the drainage site - usually, fistulas open into places of lower pressure, with shunt and volume overload of the chamber or large recipient vessel,^7^ becoming indications of the need for an approach. The 2018 update of the American College of Cardiology/American Heart Association guideline emphasizes the importance of Heart Team assessment of the feasibility and choice of approach in centers with experience in closure techniques, both surgical and percutaneous.^8^

Anatomical factors such as location (proximal or distal) influence the choice of the most appropriate method for approaching CFs. Fistulas in the distal coronary bed, as in the case of our patient, present a higher risk of complications in endovascular treatment in addition to the presence of a larger area at risk, and a surgical approach is recommended.^8^

In this particular context, a patient previously diagnosed with CAD, presenting alterations in segmental motility and the incidental discovery of a coronary-cavitary fistula during coronary angiography, presumably of congenital etiology and whose main flow was derived from the right coronary artery, manifested evident and progressive symptoms of HF, possibly resulting from the progression or coexistence of the diseases.

The case presented here is challenging in several aspects: firstly, regarding the diagnosis of the etiology of HF due to the difficulty in characterizing it using standard non-invasive imaging methods such as TTE. Although TTE has greater sensitivity for detecting flow, shunt entry, and termination sites, the size and anatomical characteristics of the fistula, in this case, had to be established by angiography, which was preferred in this case given the patient’s comorbidities and because it is considered the gold standard; regarding treatment, due to the high risk of complications during the procedures, location of the fistula, myocardium at risk, and lack of robust evidence in percutaneous treatment in patients with previous revascularization, a surgical approach was chosen; In this case, after the surgical procedure, the patient evolved favorably over the following months, with remodeling of the left chambers, a decrease in PASP from 53 to 39 mmHg and clinical improvement, currently in functional class I. Although coronary fistulas are an uncommon and often unknown etiology of HF, correction, even on late occasions, as in the case in question, can lead to an improvement in the prognosis and quality of life of patients.

## Conclusions

Coronary fistulas continue to be a difficult entity to diagnose due to their low incidence, most of the time remaining clinically asymptomatic until adulthood and, as seen in this report, being dynamic, when symptomatic, they can lead to states of hemodynamic overload that, concomitantly with other pathologies, make the management of these patients difficult.^9-11^

The scarcity of data in the literature on the subject makes it difficult not only to diagnose CFs but, above all, to treat them, especially with regard to when and how to treat them. These issues probably depend on numerous factors and should be assessed on a case-by-case basis. It is not possible to say, for example, based on the data we have available, whether the surgical approach to the fistula should have been performed at the time of the CABG in 2012 or whether it was correct to maintain a wait-and-see approach at that time. Despite this, in the absence of a control group, the prospective evaluation of the patient clearly shows the improvement presented by the patient after the procedure performed in 2023, in clinical and imaging terms, at least in the short follow-up period evaluated.
